# CellMAPtracer: A User-Friendly Tracking Tool for Long-Term Migratory and Proliferating Cells Associated with FUCCI Systems

**DOI:** 10.3390/cells10020469

**Published:** 2021-02-22

**Authors:** Salim Ghannoum, Kamil Antos, Waldir Leoncio Netto, Cecil Gomes, Alvaro Köhn-Luque, Hesso Farhan

**Affiliations:** 1 Department of Molecular Medicine, Institute of Basic Medical Sciences, University of Oslo, 0372 Oslo, Norway; hesso.farhan@medisin.uio.no; 2 Department of Integrative Medical Biology, Umeå University, 90736 Umeå, Sweden; 3 Oslo Centre for Biostatistics and Epidemiology, Faculty of Medicine, University of Oslo, 0372 Oslo, Norway; w.l.netto@medisin.uio.no (W.L.N.); alvaro.kohn-luque@medisin.uio.no (A.K.-L.); 4 University of Arizona Cancer Center, University of Arizona, Tucson, AZ 85724, USA; jrgomes07@live.com; 5 Institute of Pathophysiology, Medical University of Innsbruck, 6020 Innsbruck, Austria

**Keywords:** cell tracking, cell migration, proliferation, MATLAB, time-lapse imaging, doubling time, speed–directionality dynamics, BT549, retinal pigment epithelial cells (RPE), FUCCI

## Abstract

Cell migration is a fundamental biological process of key importance in health and disease. Advances in imaging techniques have paved the way to monitor cell motility. An ever-growing collection of computational tools to track cells has improved our ability to analyze moving cells. One renowned goal in the field is to provide tools that track cell movement as comprehensively and automatically as possible. However, fully automated tracking over long intervals of time is challenged by dividing cells, thus calling for a combination of automated and supervised tracking. Furthermore, after the emergence of various experimental tools to monitor cell-cycle phases, it is of relevance to integrate the monitoring of cell-cycle phases and motility. We developed CellMAPtracer, a multiplatform tracking system that achieves that goal. It can be operated as a conventional, automated tracking tool of single cells in numerous imaging applications. However, CellMAPtracer also allows adjusting tracked cells in a semiautomated supervised fashion, thereby improving the accuracy and facilitating the long-term tracking of migratory and dividing cells. CellMAPtracer is available with a user-friendly graphical interface and does not require any coding or programming skills. CellMAPtracer is compatible with two- and three-color fluorescent ubiquitination-based cell-cycle indicator (FUCCI) systems and allows the user to accurately monitor various migration parameters throughout the cell cycle, thus having great potential to facilitate new discoveries in cell biology.

## 1. Introduction

Motility of cells within and between tissues is a fundamental biological process that plays important roles during tissue morphogenesis, immunology, wound healing, and tumor progression [1]. To understand how cells move and what governs their migratory movements, it is often necessary to identify fluorescently labeled cells in microscopy images and track them over time. However, tracing cells that migrate, proliferate, interact, or die is a laborious and error-prone process. Historically, cell tracking has been performed by manual selection on a reference point within a cell for each time-lapse frame [2]. Such an approach is often prohibitively time-consuming and at risk of user bias due to the difficulty in manually defining cell positions and the noticeable inconsistency between different users [3]. Manual tracking is also susceptible to either overrepresenting particular angles between displacements or repeating previous *x*–*y* coordinates [4]. On the other hand, cells can be tracked automatically by leveraging prior knowledge about the morphology and motility of cells [5]. Automated tracking can be achieved by different approaches, including tracking by detection [6], deep learning-based models [7], and probabilistic prediction models [8]. Although automated tracking can provide objective migration tracks due to the elimination of human factor-related errors, it can itself generate artefacts. This is particularly the case when tracking a dense population of cells with low contrast and inadequate gap size. This may obscure biologically relevant differences between experimental settings or generate spurious results. Thus, the development of efficient tools for cell tracking with a minimum level of bias are key to obtain reliable quantitative insights from biologic experiments.

Today, a large number of tracking systems exist [9,10,11,12,13,14,15,16,17,18,19,20,21]. However, achieving the task of completeness and automation remains a challenge. Although automatic tracking eliminates the human error, it may abolish the ability to supervise, inspect, and edit the trajectories [22]. Adjusting numerous parameters to optimize performance is time-consuming and sometimes comparable with fully manual tracking. Most tracking systems do not account for cell division (Table S1, Supplementary Materials), and they either end the tracking when a cell divides or continue tracking one of the daughter cells as a continuation of the mother cell. It is, therefore, impossible with this approach to trace the history of cells and compare the migration of daughter cells and their mother cell. Moreover, this limited approach also disallows the studying of migratory changes during cell division.

On the other hand, the fluorescent ubiquitination-based cell-cycle indicator (FUCCI) is a widely used system to study the cell cycle using video microscopy [23,24,25,26]. The FUCCI system was originally developed to indicate individual cell-cycle phases with a unique fluorescent signature in a spatiotemporal manner using differentially colored fluorescent tags on two cell-cycle-regulated proteins, which are Cdt1 and Geminin [27]. Grant and colleagues enhanced the detection of gap 1 (G1)/synthesis (S) and S/G2 transitions using the proliferating cell nuclear antigen (PCNA)-interacting protein (PIP)-FUCCI construct [28]. Linking lineage tracking with FUCCI remains challenging due to the complexity of integrating the needed algorithms.

Here, we introduce CellMAPtracer, an open-source, free software tool that allows automated and supervised tracking of fluorescently labeled cells [29]. CellMAPtracer is applicable for a variety of two-dimensional (2D) cell migration assays, such as random migration and directed migration. It is capable of combining automated tracking with manual curation. It provides basic motility analysis and categorized trajectory data for deeper trajectory investigation. CellMAPtracer allows users to trace and follow individual cells throughout the course of the live imaging. This can enable the user to visualize the tracks of the descended cells and their ancestor in an interactive multigeneration plot. The obtained trajectory data can be used to precisely estimate the doubling time of the tracked cell population, as well as characterize the heterogeneity between daughter cells. Furthermore, CellMAPtracer provides the possibility to link lineage tracking with FUCCI systems, which can highlight the changes in the profiles of directionality and speed across the cell-cycle phases.

## 2. Materials and Methods

### 2.1. Cell Culture, Random Migration, and Live Cell Imaging of BT549 Cells

BT549 cells were cultured at 37 °C and 5% CO_2_ in Roswell Park Memorial Institute (RPMI) supplemented with 10% FBS (fetal bovine serum) in addition to 100 U/mL penicillin/streptomycin, which can serve as frontline therapy in the fight against bacterial contamination. To generate GFP-BT549 stably labeled cells, BT549 cells were seeded in a six-well plate at density of 1.5 × 10^5^, and, after 24 h, they were transduced with NucLight green lentivirus (Essen BioScience). Infections were carried out using a multiplicity of infection (MOI) of three transducing units per cell. On the day of infection, cells were washed with phosphate-buffered saline (PBS), and growth media containing the virus were added. Cells were incubated at 37 °C, 5% CO_2_ for 3 days with a complete medium change every 24 h. The green fluorescent protein-based fluorophores of NucLight are located within the nuclear envelope of the cell. During cell division, new NucLight protein is synthesized and transmitted to the new daughter cells. The transduced cells were sorted via fluorescence-activated cell sorting (FACS) using a BD FACSAriaTM cell sorter. For optimal tracking efficiency, a mixed population of BT549 and GFP-BT549 cells was used for the random migration assay. These cells were cocultured at a ratio of 3:1 respectively. For the two-dimensional random migration, cells from the mixed population of BT549 and GFP-BT549 cells were cultured in 96-well image-lock plates (EssenBio, 4739, Lot#17040501) for 24 h at 37 °C and 5% CO_2_. Then, cells were scanned at 10 min intervals over 3 days in Essen BioScience’s IncuCyte S3 with objective lenses 10× (numerical aperture (NA): 0.95; image resolution: 1.24 µm/pixel) using both the phase channel (HD Phase imaging in gray values) and the green channel (emission wavelength: 524 nm; excitation wavelength: 460 nm; exposure time: 200 ms). Images were collected using a Basler Ace 1920–155 um camera with a complementary metal–oxide–semiconductor (CMOS) sensor. Cellular viability was assessed throughout the course of the scanning by comparing the phase cellular morphology between BT549 and GFP-BT549 cells. Data are available at https://doi.org/10.5281/zenodo.3878526 and https://doi.org/10.5281/zenodo.4179028 (Both accessed on 20 February 2021).

### 2.2. Two-Channel FUCCI Time-Lapse Data

For the FUCCI sensor (ThermoFisher Scientific, Waltham, MA 02451, United States) experiments, the BacMam 2.0 (baculovirus) gene delivery system was utilized. The Bacmam 2.0 system was employed for its high transduction efficiency rate, as well as having minimal cytopathic effects on mammalian cells. Titration experiments of the viral particles demonstrated that 80 viral particles per cell (PPC) were suitable to yield approximately 90% transduction efficiency. The optimum expression signal was observed 36 h post transduction. For the experimental setup, H322 cells were plated at a density of 20,000 cells per well into two-well cover-glass bottom chamber slides (Thermofisher Scientific). The slides used were the Nunc Lab-Tek II chambered slides, with a culture area of 4.0 cm^2^ and a thickness of 0.16–0.19 mm. Cells were cultured in Dulbecco’s modified Eagle medium (DMEM) (Corning Cellgrow) supplemented with 10% fetal bovine serum, 100 U penicillin, and 100 mg streptomycin. Prior to the time-lapse imaging, cells were co-transduced with 80 PPC Premo geminin-GFP (G2/M reagent) and 80 PPC Premo Cdt1-RFP (G1/S reagent) following the BacMam 2.0 gene delivery protocol, and they were then allowed to incubate for 24 h at 37 °C and 5% CO_2_. Following this incubation, the cells were washed with PBS and released into complete FluroBrite DMEM (ThermoFisher) and allowed to incubate again for 12 h. The chambered slide was then transferred to a Pecon Heating Insert (Carl Zeiss), attached to the microscope stage and maintained at 37 °C at 5% CO_2_. Time-lapse images were acquired using a 20× air objective (NA: 0.95; image resolution: 0.26 µm/pixel) with a CMOS camera (Orca Flash V4.0, Hamamatsu Photonics 430-8587, Japan) on a Zeiss AxioObserver.Z1 wide-field epifluorescence. Neither photobleaching nor phototoxicity was observed in cells imaged with this protocol. Images were collected at 20 min intervals for 18 h. Data are available at https://doi.org/10.5281/zenodo.4179316 (accessed on 20 February 2021).

### 2.3. Three-Channel FUCCI Time-Lapse Data

Three-channel FUCCI time-lapse images of human telomerase reverse transcriptase (hTERT)-immortalized retinal pigment epithelial cells (RPE) were obtained from Dr. Katarzyna M. Kedziora and Prof. Jeanette Gowen Cook at the University of North Carolina, Chapel Hill, NC, USA. Data are available at https://doi.org/10.5281/zenodo.4179252 (accessed on 20 February 2021). According to the original study (Grant et al., 2018) RPE1-hTert (RPE) cells were obtained from the American Type Culture Collection (ATCC). Fluorescently labeled PCNA and PIP-FUCCI cell-cycle indicators were introduced with lentiviral vectors (Addgene 118617 and 118616). The expression construct of PIP-FUCCI consists of human Cdt1 amino acids 1–17 including the PCNA-interacting protein (PIP) degron, SV40 nuclear localization signal, epitope tag, self-cleaving peptide, and human Geminin amino acids 1–110 including both the D box and the KEN motif. Prior to imaging, cells were plated on glass-bottom plates (Cellvis) #1.5 in FluoroBrite DMEM (Invitrogen) supplemented with FBS, l-glutamine, and penicillin/streptomycin (imaging media). A Nikon Ti Eclipse inverted microscope with a Lumencore Spectra X Light Engine was used to image cells with the Nikon Perfect Focus System and a Plan Apochromat dry objective lens 40× (NA: 0.95; image resolution: 0.65 µm/pixel). Images were collected using an Andor Zyla 4.2 sCMOS detector with 12 bit resolution. All filter sets were from Chroma, YFP channel (PIP)—508 nm, 20%; 200 ms (excitation; exposure time), mTurq channel (PCNA)—440 nm, 3%; 200 ms, mCherry channel (Gem)—575 nm, 20%; 300 ms. Cells were imaged in a humidified chamber (Okolabs) at 37 °C with 5% CO_2_. No photobleaching or phototoxicity was observed in cells imaged with this protocol. The obtained movie shows RPE cells stably expressing PIP-FUCCI sensor together with fluorescently tagged PCNA protein (PCNA-mTurq2). Cells were imaged at 10 min intervals for 3 days.

### 2.4. CellMAPtracer

#### 2.4.1. Availability and Installation

CellMAPtracer was built using MATLAB (v 9.8) and can be freely obtained as (A) a standalone executable program for Microsoft Windows, macOS, or GNU/Linux, (B) a MATLAB App/Toolbox, and (C) the source MATLAB code.

(A) Standalone executable program
  This can be accessed at https://github.com/ocbe-uio/CellMAPtracer/releases/tag/v1.1 (accessed on 20 February 2021). Three assets of CellMAPtracer for Windows, Linux, and macOS versions can be found and downloaded. After downloading the version compatible with their operating system, users should decompress the file and follow the instructions in the corresponding “readme.txt”.
(B) MATLAB App
  To be able to run CellMAPtracer App within the MATLAB environment, users should follow three simple steps: (1) download the “App” folder from the CellMAPtracer repository: https://github.com/ocbe-uio/CellMAPtracer (accessed on 20 February 2021); (2) in MATLAB, go to the APPS tab and click “Install App”, find the “CellMAPtracer.mlappinstall” file, and then install it; (3) open CellMAPtracer App from the Application list in MATLAB.
(C) From the source MATLAB code
  To be able to run CellMAPtracer code, users should clone the CellMAPtracer repository from https://github.com/ocbe-uio/CellMAPtracer (accessed on 20 February 2021) and then run “CellMAPtracer_Main.m” after opening a project in MATLAB.

#### 2.4.2. CellMAPtracer Description and Workflow

CellMAPtracer is a desktop application with a graphical user interface (GUI). The utility of CellMAPtracer is enhanced through tracking and FUCCI plug-ins.

##### Tracking Single Cells

**Figure 1:**
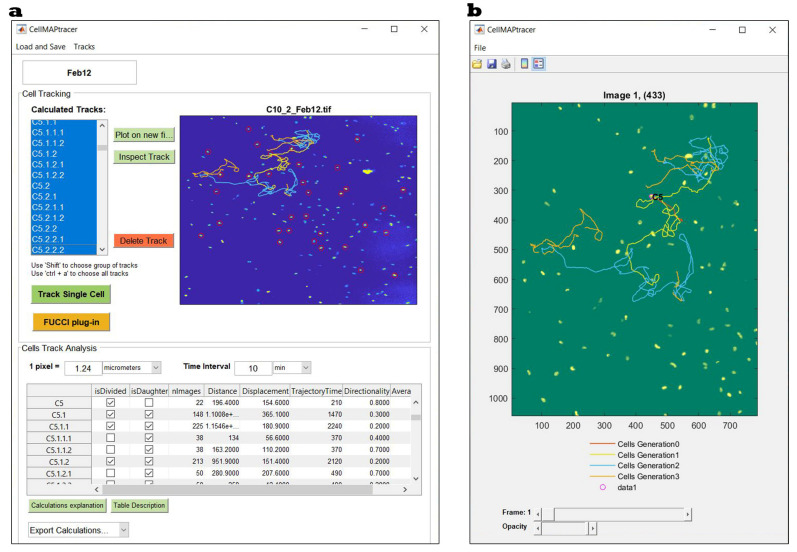
CellMAPtracer graphical user interface. (**a**) The main user interface window used to track single cells and all their descendant cells. (**b**) A representative interactive multigeneration trajectory plot of a cell (orange) and its descendant daughters (yellow) and granddaughters (blue).

CellMAPtracer is capable of loading multi-TIFF stacks (8 and 16 bits) of spatiotemporal live cell images as input for tracking (Figure 1a). The output is an interactive multigeneration trajectory plot (Figure 1b) and five categories of trajectory data. The five categories include all cells, dividing cells, nondividing cells, daughter cells, and dividing daughter cells. Each of these contains two spreadsheets. The first sheet contains the measurements of cell migration parameters such as the total distance, displacement, directionality, and speed (Figure S1, Supplementary Materials). The second sheet contains the *x*–*y* coordinates of tracked cells in the corresponding category. The purpose of the categorization is to enable users to easily plot the cell migration statistics without the need for any programming skills. Such plots can help highlight the migratory phenotype of cells in each category and draw conclusions about the doubling time, the heterogeneity of daughter cells, and speed–directionality dynamics prior and through cell division.

Currently, CellMAPtracer is based on two classical detection approaches for nucleus segmentation. The first approach uses a paradigm called tracking by detection. It relies on a fluorescence detector to initialize, adjust, reinitialize, supervise, and terminate a tracker [30]. CellMAPtracer analyzes the frame-to-frame position of a target cell. For each frame, contrast-limited adaptive histogram equalization is used to separate nuclei from the background by converting grayscale images into binary images. All above-threshold contiguous regions are considered nuclear objects taking into account the spatial characteristics of segmentation. The second approach of cell segmentation is the watershed transformation. This finds ”watershed ridge lines” in an image and treats the image as a surface where light pixels of the nuclei represent high elevations and dark pixels of the background represent low elevations [31]. This fast and intuitive method allows separating close nuclei from each other regardless of the similarity degree in the signal intensity [32]. As a result, accurate, instant segmentation is generated. After nucleus detection, the position of each nucleus is determined by finding its center of mass, which is calculated on the basis of all the pixels in the particle having the same intensity. Positions of all nuclei within the field of view are compared with the position of the tracked cell on the last frame. To find the new cell position, the algorithm calculates the distances between the last position of the target cell nucleus and current position of each other nucleus. The position with minimum distance is set as the new position of the tracked cell (Figure S2, Supplementary Materials). To save computational time, the local features are computed only within a fraction of the image defined by the interactive slider in the CellMAPtracer tracking window. MATLAB programmers can fully edit, extend, and add more tracking algorithms to CellMAPtracer.

We designed CellMAPtracer to allow cell tracking to be done automatically and monitored in a stepwise manner (Figure S3, Supplementary Materials). Tracking errors can distort the cell trajectory results. For that reason, we designed the CellMAPtracer interface to include multiple features for importing, highlighting cell division, inspecting, and correcting existing tracked cells/nuclei to reach near 100% tracking accuracy. In case the algorithm mistakenly tracks the target cell or switches to another cell, the user can manually correct the tracking by pausing the automated tracking and clicking on the correct position on the current frame. The corrected position will be corrected further if the “use center of mass after manual correction” button is activated. Otherwise, the exact position selected by the user will be recorded. In case the intensity of the target cell/nucleus is very low, the “use center of mass after manual correction” button should be deactivated. During the course of tracking, the migratory cell might undergo cell division. CellMAPtracer allows independently marking cell divisions and lineage tracing of all descendant cells. From the perspectives of intensity and morphology, cell division is a very dynamic process. The naked eye can, in most cases, recognize the occurrence of cell division. However, automated cell division detection, which mainly benefits from the representational power of deep learning models, requires enormous computational power and training copious amounts of data [33]. To handle such difficulties, when the user notices a division event, a single-click user intervention is needed. This simple intervention initiates tracks for the new daughter cells. There is no limitation to the number of marked divisions. Therefore, CellMAPtracer can optimally handle long-term live imaging experiments.

##### FUCCI Plug-In

**Figure 2:**
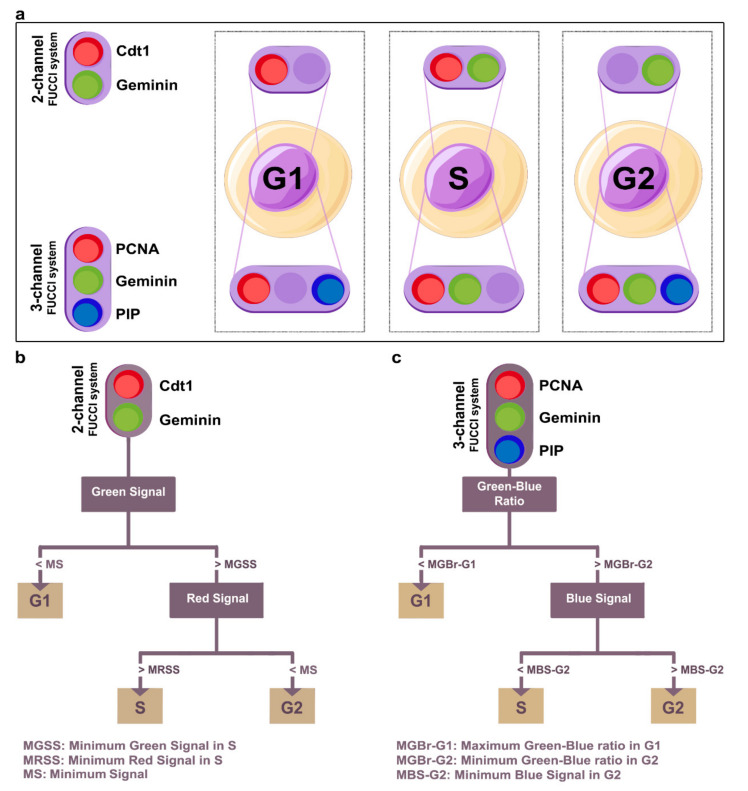
A description of the phase prediction methods of CellMAPtracer fluorescent ubiquitination-based cell-cycle indicator (FUCCI) plug-in. (**a**) Schematic representation of FUCCI labeling across the cell-cycle phases. (**b**) The mechanistic prediction process of the cell-cycle phase in a two-channel FUCCI system. (**c**) The mechanistic prediction process of the cell-cycle phase in a three-channel FUCCI system.

The CellMAPtracer FUCCI plug-in enables users to profile the fluorescent signals of FUCCI-expressing cells in 2–3-channel systems. It detects the cell-cycle phase at any given time point throughout the course of the tracking. The input of the FUCCI plug-in is a multi-TIFF stack in red/green/blue (RGB) format of spatiotemporal live cell images which should be associated with the outcome of the tracking outcome of CellMAPtracer for the corresponding multi-TIFF stacks (8 and 16 bits) (Figure S4, Supplementary Materials). After loading the needed files and selecting the cell-cycle detection method, the FUCCI phase algorithm automatically implements an internal RGB normalization. Users can inspect the normalized and the raw signals and monitor the detection of the cell-cycle phases with the possibility of correcting the detection. This can be done by selecting the phase and placing a number in the “from frame” and “till frame” fields then clicking on correct. The efficiency of the cell-cycle detection depends on the purity of the unicellular signal source. The signal detection field should not exceed the cell diameter, which can vary within the population of imaged cells. CellMAPtracer-FUCCI enables users to select the overall cell diameter on the front window, and this will be used for all cells. However, that value can be adjusted for particular cells when it is needed. Currently, the CellMAPtracer FUCCI plug-in provides two methods for the detection of cell-cycle phases:

(A) Two channel system
  The RGB multi-TIFF stacks should be formatted with the red channel corresponding to Cdt1 and the green channel corresponding to Geminin (Figure 2a). Cdt1 and Geminin have opposing effects on DNA replication and show inverse oscillation patterns during the cell cycle. Cdt1 protein (red signal) peaks in G1 phase and then declines in S phase before becoming high again at the M/G1 transition [34]. In contrast, Geminin (green signal) is destabilized during G1 phase but accumulates during S and G2 phases [35]. The cell-cycle detection approach is based on three parameters: the minimum signal (MS), the minimum green signal in S phase (MGSS), and the minimum red signal in S phase (MRSS). The default values for MS, MGSS, and MRSS are set to 0.1 with the possibility of being adjusted by users. Figure 2b demonstrates the mechanism for determining the cell-cycle phase in a two-channel system.
(B) Three-channel system
  The RGB multi-TIFF stacks should be formatted with the red channel corresponding to PCNA, the green channel corresponding to Geminin, and the blue channel corresponding to PIP (Figure 2a). PIP-FUCCI is a degradation-based biosensor of cell-cycle phases [28]. It includes two fluorescently tagged sensors—the PIP degron (Cdt1-PIP fragment) and Geminin1-110 fragment. PIP-mVenus is a direct indicator of replication as its dynamics is physically coupled to the PCNA presence at the replication forks. On the other hand, Gem1-110 (fused to mCherry fluorescent protein), as an antigen presenting cell (APC)-Cdh1 target, is an indirect sensor of the G1/S transition and is present in S and G2 phases of the cell cycle. The approach to detecting the cell-cycle phases is based on three parameters: the maximum green-blue ratio in G1 phase (MGBr-G1), the minimum green-blue ratio in G2 phase (MGBr-G2), and the minimum blue signal in G2 phase (MBS-G2). The default values for MGBr-G1, MGBr-G2, and MBS-G2 are set to 0.5, 0.7, and 0.1, respectively, with the possibility to adjust the values by the user. Figure 2c demonstrates the mechanism for determining the cell-cycle phase in a three-channel system.

### 2.5. Trajectory Analysis

Basic trajectory analyses, including total distance, displacement, directionality, and speed, are automatically done by CellMAPtracer. Excel and many online tools can be used to visualize the statistical results through boxplots, scatter plots, and bar plots. A list of suggested online visualization tools is available on the CellMAPtrace readme page: https://github.com/ocbe-uio/CellMAPtracer (accessed on 20 February 2021). All the trajectory analyses in the study were carried out in the statistical programming language R [36]. All the needed codes for the trajectory analysis with tutorials are available at the CellMAPtracer Wiki page: https://github.com/ocbe-uio/CellMAPtracer/wiki (accessed on 20 February 2021).

## 3. Results

### 3.1. Tracking and Analyzing Human Breast Cancer Cells

**Figure 3:**
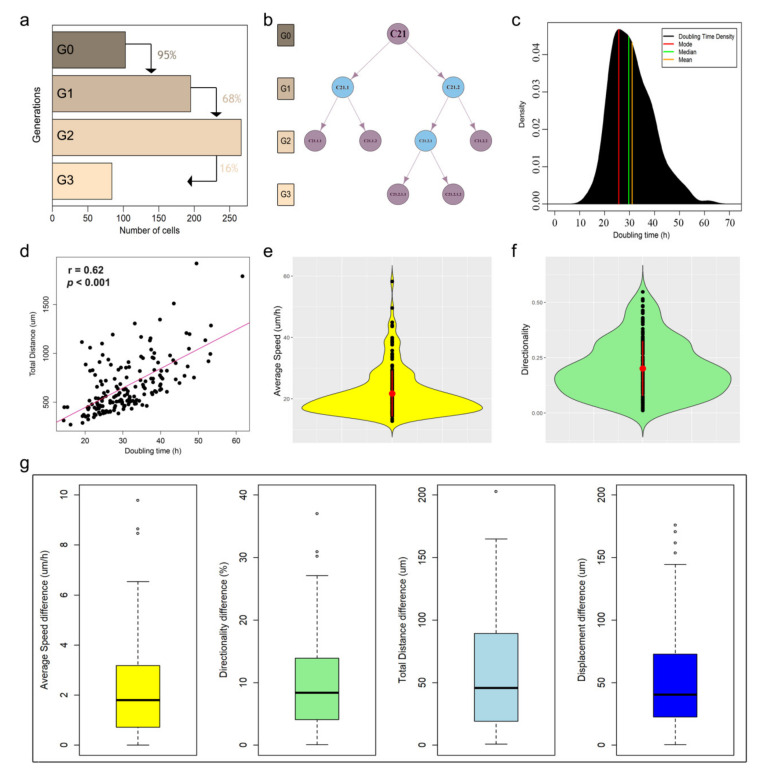
Characterizing the trajectory movement of BT549 cells. (**a**) Bar plot showing number of tracked cells per generation. (**b**) Lineage tree of cell #21 and its descendants; blue vertices refer to dividing daughter cells. (**c**) Density plot of the doubling time of BT549 cells (*n* = 175). (**d**) Correlation between BT549 doubling time and total migration distance (Spearman’s rank correlation coefficient = 0.6). (**e**,**f**) Violin plots showing the median and the interquartile range of the average speed and directionality of BT549 cells. (**g**) Boxplots showing the heterogeneity among BT549 daughter cells (*n* = 121 pairs) as a function of four migration parameters: total distance, displacement, directionality, and average speed.

As a proof of concept, we used CellMAPtracer to track and analyze human breast cancer cells migrating randomly in 2D space. We used the triple-negative breast cancer cell line of BT549, stably expressing nuclear green fluorescent protein (GFP). From the first frame of three multi-TIFF 8 bit image stacks [37], a total of 103 cells were randomly selected to be tracked. Other cells were also tracked but they were manually excluded due to fluorescence intensity issues (i.e., the fluorescence intensity was too low) or early disappearance from the scanning field. These 103 ancestor cells (referred to as G0) and their descendants (referred to as G1, G2, and G3) were followed during 72 h of live imaging. All the calculated tracks are available at https://github.com/ocbe-uio/CellMAPtracer/tree/master/Data (accessed on 20 February 2021). At the end of the tracking course, a total of 648 cells distributed over four generations were tracked (Figure 3a). From all tracked cells (ancestors and descendants), 42% underwent cell division. Furthermore, 27% of all tracked cells showed complete cell-cycle phases. Such cells were referred to as dividing daughter cells, which were generated from parent cells and underwent a second round of division themselves. Users can easily plot the lineage tree of any target ancestor cell and its descendants. Lineage trees are hierarchical flowchart-like structures involving successive binary divisions and consisting of arrows and vertices. Arrows refer to cell division, whereas vertices refer to cells (Figure 3b). The trajectory time of the dividing daughter cells gives a precise estimation of the doubling time. In our case, the doubling time of BT549 cells (*n* = 175) averaged 31.1 ± 8.5 h with a median of 30.2 h and a mode of 24.7 h (Figure 3c). Correlation analysis showed a negligible correlation between the doubling time and both the speed and the directionality of movement. On the other hand, the doubling time was moderately correlated (*r* = 0.6) with the total migration distance (Figure 3d).

BT549 dividing daughter cells were characterized by an average speed of 21.7 ± 7.5 µm/h and a directionality of 0.2 ± 0.1 (Figure 3e,f). Next, we show how CellMAPtracer enables users can gain insight into the heterogeneity between daughter cells. The median trajectory time of all daughter cells (*n* = 544) was 25.5 h (Figure S5a, Supplementary Materials). For a meaningful comparison, the considered trajectory time should be long enough and comparable between the two daughter cells. Here, on the basis of the distribution of the trajectory time of daughter cells, we selected the cells that had a minimum trajectory time of 10 h with no trajectory time difference larger than 3 h between the two daughter cells. A total of 121 pairs of daughter cells were used as input for the heterogeneity analysis. Interestingly, the two daughter cells showed relatively different trajectory measure values (Figure 3g). We also assessed the synchrony of the division of daughter cells by comparing 71 pairs. We noticed that the majority daughter cells did not divide at exactly the same time, but with some difference in the range of 5 h. Only 10% of the daughter cells divided with precise synchronization. The mode time difference was 2 h. The mean time difference was 5.7 h with a standard deviation of 5.5 h (Figure S5b, Supplementary Materials). Thus, CellMAPtracer allows users to create lineage histories of cells and their daughters and compare how two daughter cells behave.

**Figure 4:**
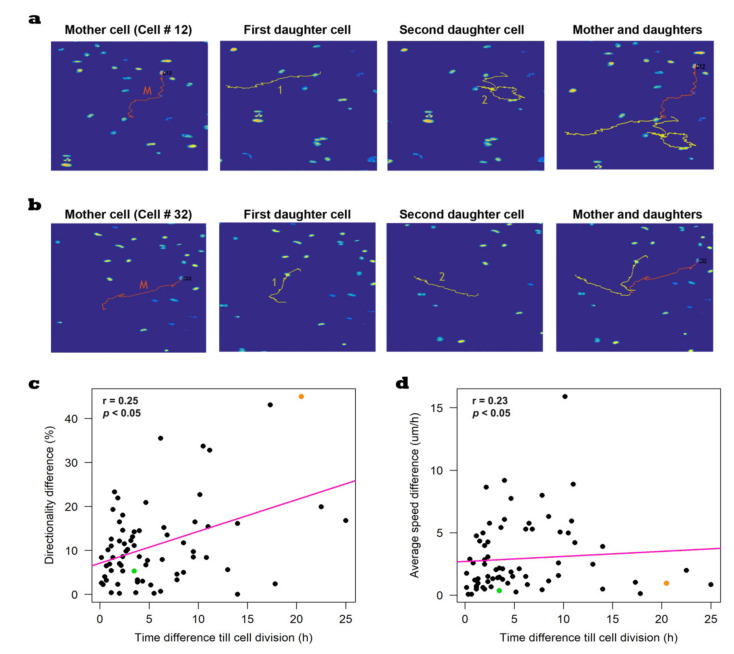
Representative examples of the heterogeneity between BT549 daughter cells. (**a**) An example of a cell (cell number 12 in red) with heterogeneous daughter cells (yellow). (**b**) An example of a cell (cell number 32 in red) with relatively homogeneous daughter cells (yellow). (**c**,**d**) Weak correlation between the time difference till cell division with both the directionality difference between daughter cells (Spearman’s rank correlation coefficient = 0.25) and the average speed difference (Spearman’s rank correlation coefficient = 0.23) between daughter cells. In the scatter plots, the orange dot represents the difference between daughter cells of cell number 12, whereas the green dot represents the difference between daughter cells of cell number 32.

We showcase examples of daughter cells with heterogeneous tracks from the directionality point of view but not speed (Figure 4a), as well as daughter cells with relatively homogeneous tracks from directionality–speed perspectives (Figure 4b). The correlation analysis shows that the time difference until cell division between daughter cells was weakly correlated with both the directionality difference (*r* = 0.25) and the average speed difference (*r* = 0.23) between the daughter cells (Figure 4c,d).

### 3.2. Tracking and Analyzing Human Epithelial Cells Expressing FUCCI

**Figure 5:**
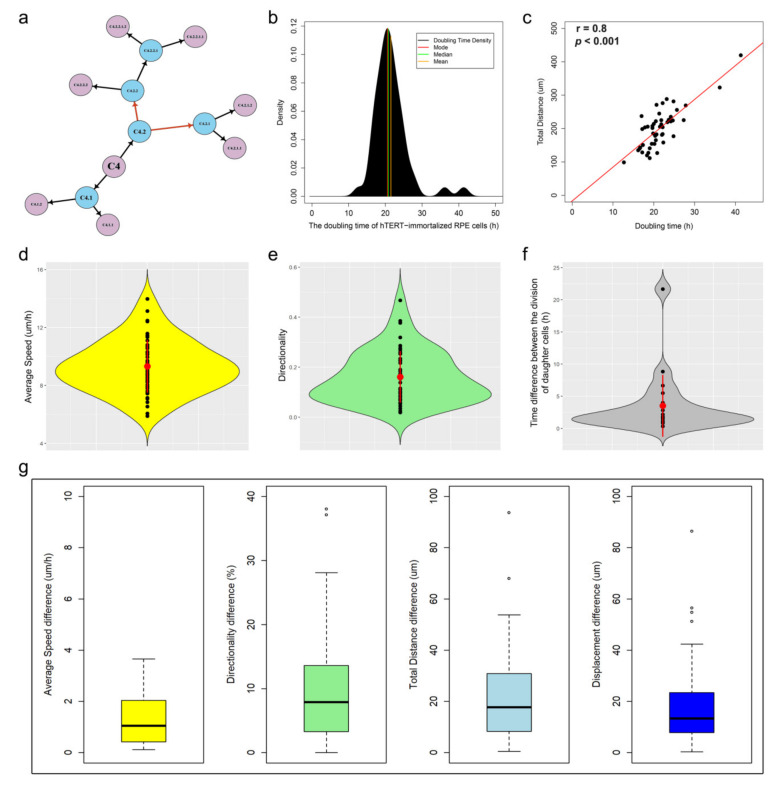
Characterizing the trajectory movement of retinal pigment epithelial (RPE) cells. (**a**) Lineage tree of cell #4 and its descendants. The length of the arrows is associated with the time till cell division. Red arrows show a deviation in the synchronization between daughter cells. (**b**) Density plot of the doubling time of RPE cells (*n* = 48). (**c**) Correlation between RPE doubling time and their total distance (Spearman’s rank correlation coefficient = 0.8). (**d**,**e**) Violin plots showing the median and the interquartile range of the average speed and directionality of RPE cells. (**f**) Violin plot showing the time difference between divisions of RPE daughter cells (*n* = 20 pairs). (**g**) Boxplots showing the heterogeneity between of RPE daughter cells (*n* = 33 pairs) according to four migration measures of total distance, displacement, directionality, and average speed.

To showcase the features of CellMAPtracer-FUCCI, we characterized the migratory movements of hTERT-immortalized retinal pigment epithelial cells (RPE) expressing PIP-FUCCI. From the first frame of multi-TIFF 8 bits image stacks [38], a total of 12 cells were manually selected to be tracked. Other cells were also tracked but they were manually excluded due to fluorescence intensity issues or early disappearance from the scanning field. These 12 cells and their descendants were followed during 72 h of live imaging. At the end of the tracking course, a total of 132 cells were tracked. From all tracked cells (ancestors and descendants), 46% underwent cell division with 48 dividing daughter cells. Figure 5a demonstrates the lineage tree of a cell and its descendants. The length of the arrows is associated with the time till cell division. Red arrows show a deviation in the synchronization between daughter cells. The doubling time of RPE-PIP-FUCCI cells averaged 21.52 ± 4.8 h with a median of 20.7 h and a mode of 20.6 h (Figure 5b). Correlation analysis showed strong correlation (*r* = 0.8, *p* < 0.001) between the doubling time and the total distance of movement (Figure 5c), as was the case with BT849 cells. However, the doubling time was significantly correlated neither with directionality of movement (*r* = −0.27, *p* = 0.06) nor with average speed (*r* = 0.1, *p* = 0.6). These dividing daughter cells were characterized by an average speed of 9.3 ± 1.8 µm/h and a directionality ratio of 0.16 ± 0.1 (Figure 5d,e). To investigate the time difference between the division of daughter cells, only daughter cells with both cells undergoing cell division were included in the analysis (*n* = 20 pairs). The average time difference between the division of daughter cells was 3.5 ± 4.8 h (Figure 5f). To characterize the heterogeneity between the RPE daughter cells, we analyzed 34 pairs of daughter cells. For a meaningful comparison, these cells were selected in such way to have a minimum trajectory time of 6 h and no trajectory time difference larger than 2.5 h between the two daughter cells. The two daughter cells showed relatively small median differences (Figure 5g) regarding speed (1.05 µm/h), directionality (7.90%), displacement (13.40 µm), and total distance (17.70 µm).

**Figure 6:**
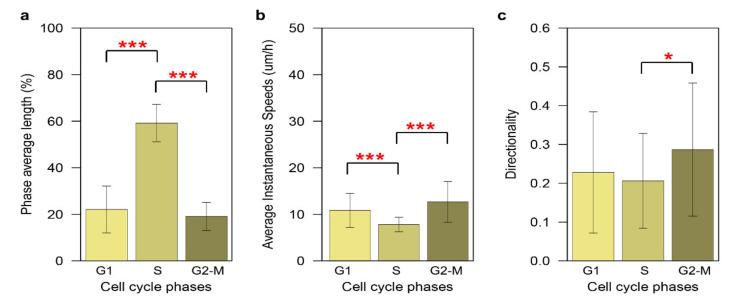
The speed–directionality dynamics across cell-cycle phases in RPE cells. (**a**) The duration distribution of each of the cell-cycle phases across all the dividing daughter cells (*n* = 48). (**b**) Instantaneous speed dynamics across cell-cycle phases. (**c**) Directionality dynamics across cell-cycle phases. Statistical significance was determined using the Kruskal–Wallis rank sum test, followed by pairwise comparisons using the Wilcoxon rank sum test (* *p* < 0.05, *** *p* < 0.001).

In order to study the speed–directionality dynamics across the entire cell cycle (G1, S, and G2/mitosis (M)), we analyzed the FUCCI signals through the trajectory paths of RPE dividing daughter cells using CellMAPtracer-FUCCI. First, we checked the duration distribution of each of the cell-cycle phases across all the dividing daughter cells (*n* = 48). The S phase constituted 59% of the entire cell-cycle duration comparing to 22% for G1 and 19% for G2/M (Figure 6a). The distribution of the cell-cycle phases of RPE cells matches with the original studies [28,39]. The G2/M phase refers to a particular period of cell division, which includes the G2 phase and mitosis. From a cell migration point of view, the G2/M phase is highly important due to the disassembly of the Golgi apparatus [40,41]. Intactness of the Golgi is known to play an important role in regulating cell migration [42,43,44,45]. The speed–directionality dynamics showed a significant decrease in the instantaneous speed during S phase 7.8 ± 1.6 µm/h compared to 10.8 ± 3.7 µm/h during G1 and 12.6 ± 4.4 µm/h during G2/M (Figure 6b). Cells seemed to maintain same directionality degree during G1 and S (0.23 ± 0.2 and 0.21 ± 0.2, respectively), but this significantly increased (0.29 ± 0.2) during G2/M (Figure 6c).

**Figure 7:**
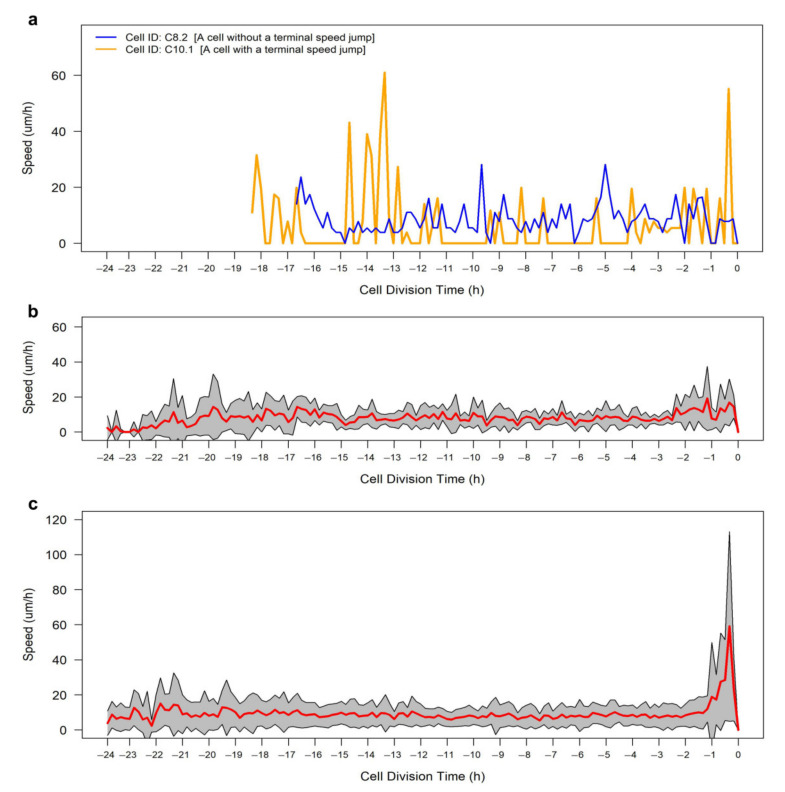
Speed profile of RPE cells across cell-cycle phases. (**a**) Examples of RPE cells without (C8.2) and with (C10.1) a terminal speed jump. (**b**) Speed profile of cells (*n* = 13) without a terminal speed jump. (**c**) Speed profile of cells (*n* = 35) with a terminal speed jump. Red lines show the average instantaneous speeds, whereas the gray shaded areas show the standard deviation.

During the tracking process, we observed a phenomenon that, to the best of our knowledge, has not been previously described and that we propose to call the “terminal speed jump” (Video S1, Supplementary Materials). The trajectory analysis of the tracked cells shows that 81.4% of the RPE cells exhibited one or more instantaneous dramatic changes in speed within the last hour prior to generating daughter cells (Figure 7). A cell is marked as undergoing a terminal speed jump if it shows one or more deviating instantaneous speeds, during the last hour of G2/M phase, with at least a 2.5-fold increased difference compared to the average instantaneous speeds during the last 2 h prior to generating daughter cells. Moreover, that instantaneous speed should be at least threefold the neighbor’s instantaneous speeds during the last hour of G2/M phase. The biological basis for this should be investigated in future work.

### 3.3. Evaluation of the Tracking Accuracy

**Figure 8:**
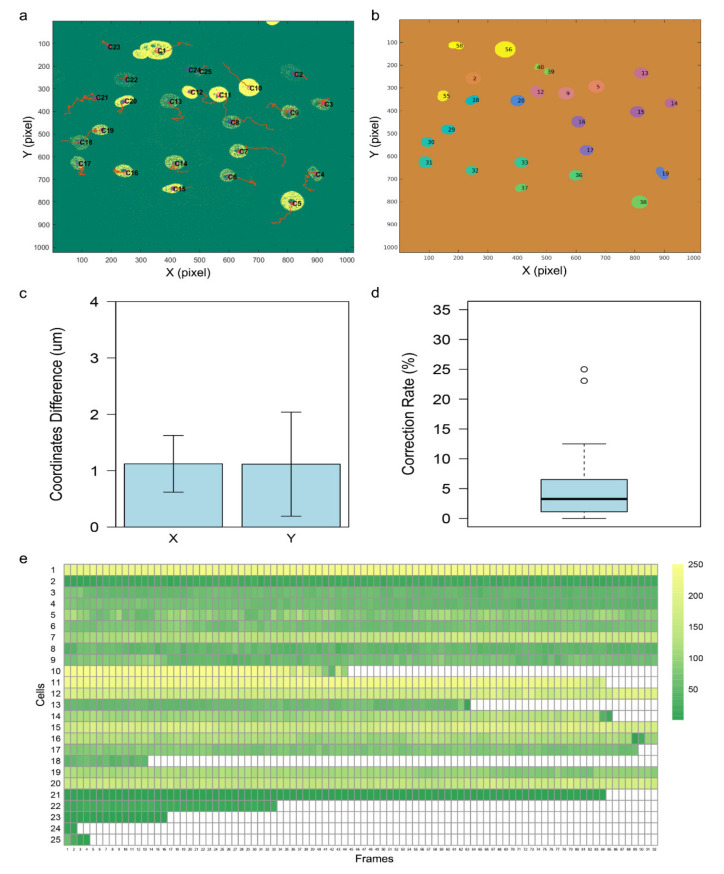
Tracking accuracy of CellMAPtracer. (**a**) Tracking outcome of CellMAPtracer over the Flue-N2DH-GOWT1_02_GT dataset. (**b**) The reference annotation of the Flue-N2DH-GOWT1_02_GT dataset computed using an inhouse code. (**c**) Location error plot; bars represent the mean coordinates difference between the trajectories generated by CellMAPtracer and the true coordinates on the *x* and *y* axes based on the ground truth (*n* = 25). (**d**) Boxplot showing the correction rates (manual intervention rates) of all tracked cells. (**e**) Intensity heatmap showing the intensity profiles of all the tracked cells.

To evaluate the performance of our method, we assessed the tracking accuracy of CellMAPtracer using a well-known metric in multiobject tracking evaluation: the location error [16,46]. The location error was computed by examining the number of cells that can be detected in the first frame and followed across all the frames, where the *x* and *y* coordinates of the centroids of the cells are reported and evaluated as a comparison with the ground truth. For that purpose, we used a dataset of GFP-GOWT1 mouse stem cells [47], named “Flue-N2DH-GOWT1_02_GT”, obtained from http://celltrackingchallenge.net (accessed on 20 February 2021), which provides a reference annotation according to consensual or majority opinion of several human experts [48]. The reference annotation of the target dataset is available in the TRA folder in the 02_GT at http://data.celltrackingchallenge.net/training-datasets/Fluo-N2DH-GOWT1.zip (accessed on 20 February 2021). The evaluation process consisted of three steps. Firstly, we tracked all the cells that appear in the first frame (Figure 8a) and exported the trajectory coordinates. Secondly, we detected the identifiers (IDs) of the cells (Figure 8b) and computed the true coordinates on the basis of the gold tracking truth using inhouse code available at https://github.com/ocbe-uio/CellMAPtracer/tree/master/Data/Evaluation (accessed on 20 February 2021). After extracting the true *x* and *y* coordinates, we calculated the location error by computing the coordinate difference between those generated by CellMAPtracer and the true *x* and *y* coordinates per cell per frame (Data S1, Supplementary Materials). The mean difference in the *x* coordinates was 1.1 ± 0.5 µm, whereas the mean difference in the *y* coordinates was 1.1 ± 0.9 µm (Figure 8c). Furthermore, we computed, for every cell, the correction rate (manual intervention rate), which is the number of corrected frames divided by the total number of frames. The median correction rate was 3.26% (Figure 8d). To inspect the ability of CellMAPtracer to trace cells regardless of their fluorescence intensity, we extracted the intensity values for each cell across all the frames using the CellMAPtracer FUCCI plug-in to plot the intensity profiles (Figure 8e). The intensity heatmap shows that some cells (dark-green bars) tracked with CellMAPtracer exhibited very low intensity. As a result, CellMAPtracer achieved very high accuracy since none of the individual coordinate differences exceeded 20 pixels (4.8 µm) [16]. On the other hand, the rate of mostly tracked trajectories by CellMAPtracer over the Flue-N2DH-GOWT1_02_GT was only 42% because, currently, CellMAPtracer enables users to select the cells only from the first frame. A total of 59 cells were identified on the basis of the reference annotation with only 25 cells from the first frame. All 25 cells were tracked completely and accurately by CellMAPtracer. In order to evaluate the performance of CellMAPtracer in a dense population, we relied on a multi-TIFF stack of densely populated BT549 cells [49]. This population contains many cells that are challenging to track (Figure S6, Supplementary Materials). With CellMAPtracer, we tracked about 200 cells. The entire tracking procedure took about 3 h using a laptop (Intel Core i5-8300H).

## 4. Discussion

CellMAPtracer is an open-source and easy-to-use software for tracking and extracting trajectory data of fluorescently labeled cells through a user-friendly GUI. CellMAPtracer was designed with the aim of providing users with highly efficient tracking of migratory proliferating cells over multiple days through supervising, inspecting, and correcting the tracking data in an enjoyable manner. Moreover, CellMAPtracer can be used in a fully automated manner at the single-cell level. As a proof of concept, breast cancer cells were scanned for 3 days. Over 100 cells were randomly tracked. To better evaluate and understand the resulting tracks, CellMAPtracer offers options to visualize and extract the resulting trajectory data. Users can interactively visualize any tracked cell and its descendants and compare the values of their migration measures and trajectory data. Such comparisons give a quick and precise characterization of the tracked cells. In particular, they allow unambiguously estimating the doubling time of the studied cells. The literature shows a wide spectrum of doubling time for BT549 cells, from 25.5 h [50] to 51 h [51] to 3.7 days [52]. A classical way of computing the doubling time uses initial and final cell counts in cultures and assumes exponential growth. CellMAPtracer, instead, enables user to get a real-time estimation of the doubling time directly from the trajectory time of the dividing daughter cells (Figure 3c).

CellMAPtracer can also shed light on the synchronization degree in terms of the division and migration between daughter cells. Our results for BT549 and RPE cells showed that the majority of daughter cells did not follow the same migratory pattern and only small proportion of daughter cells divided synchronously. The speed–directionality dynamics across the cell cycle is an enigma. The categorical output of CellMAPtracer FUCCI plug-in enables users with basic programming skills to gain extra insights into the speed–directionality dynamics of dividing cells. Users can get doubling time data and compare them to lengths of cell-cycle phases and cell migration for a large number of cells. Our results showed that RPE cells expressing PIP-FUCCI had significantly higher directionality and average instantaneous speed during the G2/M phase (Figure 6). During tracking, CellMAPtracer can help users detect unusual phenomena. We noticed an unusual phenomenon in RPE and BT549 cells, which we referred to as the terminal speed jump. A terminal speed jump was observed in 81.4% of the dividing RPE cells (Figure 7) and 60.5% of the dividing BT549 cells (Figure S7, Supplementary Materials). We also observed in BT549 cells the phenomenon of multi-daughter cell division, where more than two daughter cells are generated (Video S2, Supplementary Materials). Multi-daughter cell division is known to occur in aneuploid cancer cells [53,54]. The three or more daughter cells are usually unevenly sized [53]. While multi-daughter divisions are known, the terminal speed jump may be interesting to investigate. We can only speculate what this phenomenon might be attributed to. As cells progress toward and through mitosis, they are known to become rounder. This rounding is due to inactivation of the small GTPase Rap1 and consequently weakening or disassembling focal adhesions [55,56,57]. The reduced adhesiveness might enable cells to dislocate and increase their speed for a very short period of time. The correlation of focal adhesion disassembly with the terminal speed jump is an interesting area for future investigation.

Currently, CellMAPtracer is applicable only for fluorescently labeled cells in 2D cell migration assays and enables users to select the cells only from the first frame, in addition to the needed user interaction to detect cell divisions. Importantly, the features of CellMAPtracer can be expanded in future work to have CellMAPtracer as an ImageJ plug-in to include the possibility of selecting target cells from any frame, in addition to inspecting the four-color FUCCI system [58] or enabling users to internally run all the trajectory analyses presented in this study without the need for using external tools to run the analysis. At the moment, we provide all the needed codes for the trajectory analysis as R scripts with tutorials so that users can track cells and analyze their trajectories.

## 5. Conclusions

In this paper, we proposed a semiautomated supervised method to track and analyze the trajectory movements of fluorescently labeled cells, in addition to estimating the cell-cycle phase of FUCCI cells. With CellMAPtracer, it is straightforward to trace individual migratory proliferating cells in long-term cultures and follow their descendants efficiently using a user-friendly graphical interface without any need for coding or programming skills. The lineage tracing of all descendant cells and their ancestors allows a better computation of the doubling time and understanding of the heterogeneity of daughter cells, in addition to characterizing the speed–directionality dynamics prior to and through cell division in FUCCI-expressing cells. We showed that our tool achieves high tracking accuracy independently of the fluorescence intensity. Additionally, we confirmed the usefulness of CellMAPtracer in providing insight into dynamic single-cell behaviors using breast cancer cells and hTERT-immortalized retinal pigment epithelial cells.

## Data Availability

CellMAPtracer is an open-source multiplatform tracking system whose source code, data, and tutorials are deposited in a GitHub repository at https://github.com/ocbe-uio/CellMAPtracer. Moreover, the multi-TIFF image stacks are available at the following DOIs: 10.5281/zenodo.3878526 (BT549 sparse population), 10.5281/zenodo.4179028 (BT549 dense population), 10.5281/zenodo.4179316 (H322-FUCCI), 10.5281/zenodo.4179252 (REP-FUCCI), and 10.5281/zenodo.4485316 (Flue-N2DH-GOWT1_02_GT).

## Supplementary Materials

The following are available online at https://www.mdpi.com/2073-4409/10/2/469/s1: Figure S1. A schematic diagram elucidating how the migration measures are calculated; Figure S2. A schematic diagram explaining the tracking paradigm; Figure S3. CellMAPtracer workflow; Figure S4. CellMAPtracer FUCCI plug-in workflow; Figure S5. Trajectory time of BT549 daughter cells; Figure S6. Evaluating the performance and accuracy of CellMAPtracer; Figure S7. Speed profile across the cell-cycle phases; Video S1. Terminal speed jump in RPE cells; Video S2. Multi-daughter cell division in BT549 cells; Table S1. A comparative analysis of CellMAPtracer vs. other tracking tools; Data S1. Results of evaluating the tracking accuracy of CellMAPtracer.
