# Supplementary material for: CellMAPtracer: A User-Friendly Tracking Tool for Long-Term Migratory and Proliferating Cells Associated with FUCCI Systems
Source: Cells. 2021 Feb 22;10(2):469. doi: 10.3390/cells10020469 (PMC7927118; doi:10.3390/cells10020469)
Supplement: Supplementary file 1 [file cells-10-00469-s001.zip › CellMAPtracer Supplementary Materials/CellMAPtracer Supplementary Materials.pdf]

CellMAPtracer: A user-friendly tracking tool for long-term migratory and proliferating cells associated with FUCCI systems

Salim Ghannoum[1*], Kamil Antos[2*], Waldir Leoncio Netto[3], Cecil Gomes[4], Alvaro Köhn-Luque[3] and Hesso Farhan[1]

1 Department of Molecular Medicine, Institute of Basic Medical Sciences, University of Oslo, Norway.
2 Department of Integrative Medical Biology, Umeå University, Umeå, Sweden.
3 Oslo Centre for Biostatistics and Epidemiology, Faculty of Medicine, University of Oslo, Norway.
4 University of Arizona Cancer Center, University of Arizona, Tucson, Arizona, USA
* Correspondence: salim.ghannoum@medisin.uio.no; kamil.antos@umu.se; Tel.: +46 76 5770129 (S.G.)

Supplementary Materials

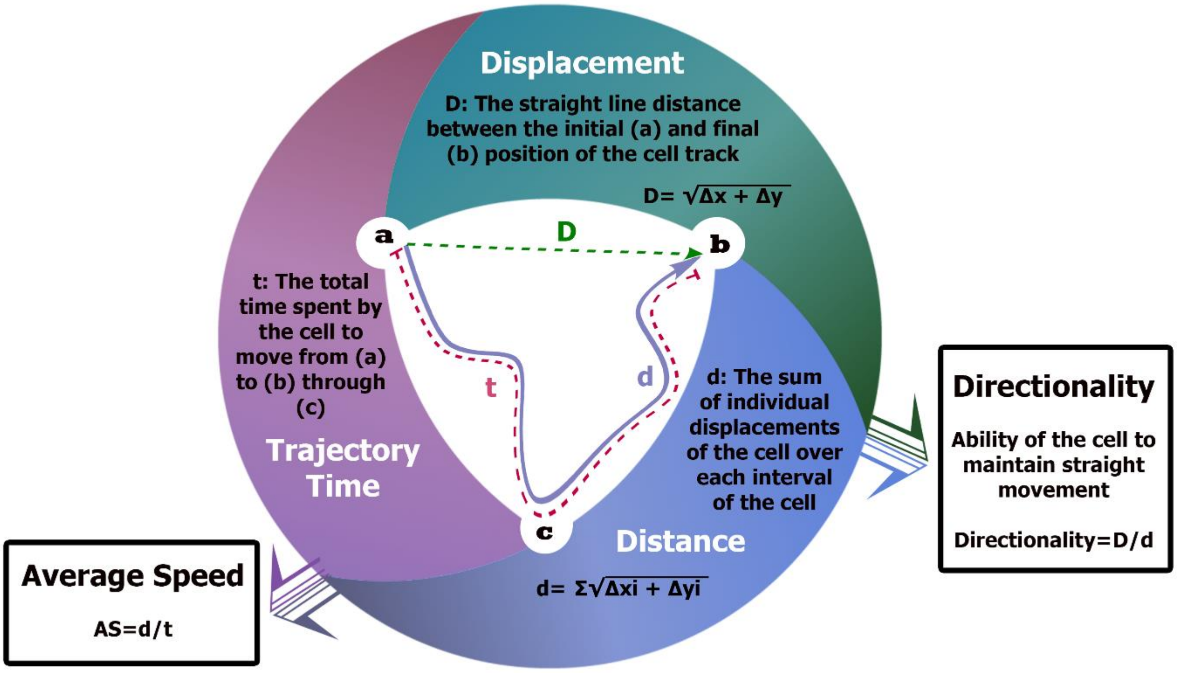

Figure S1. A schematic diagram elucidates how the migration measures are calculated in addition to description of the quantification acquired.

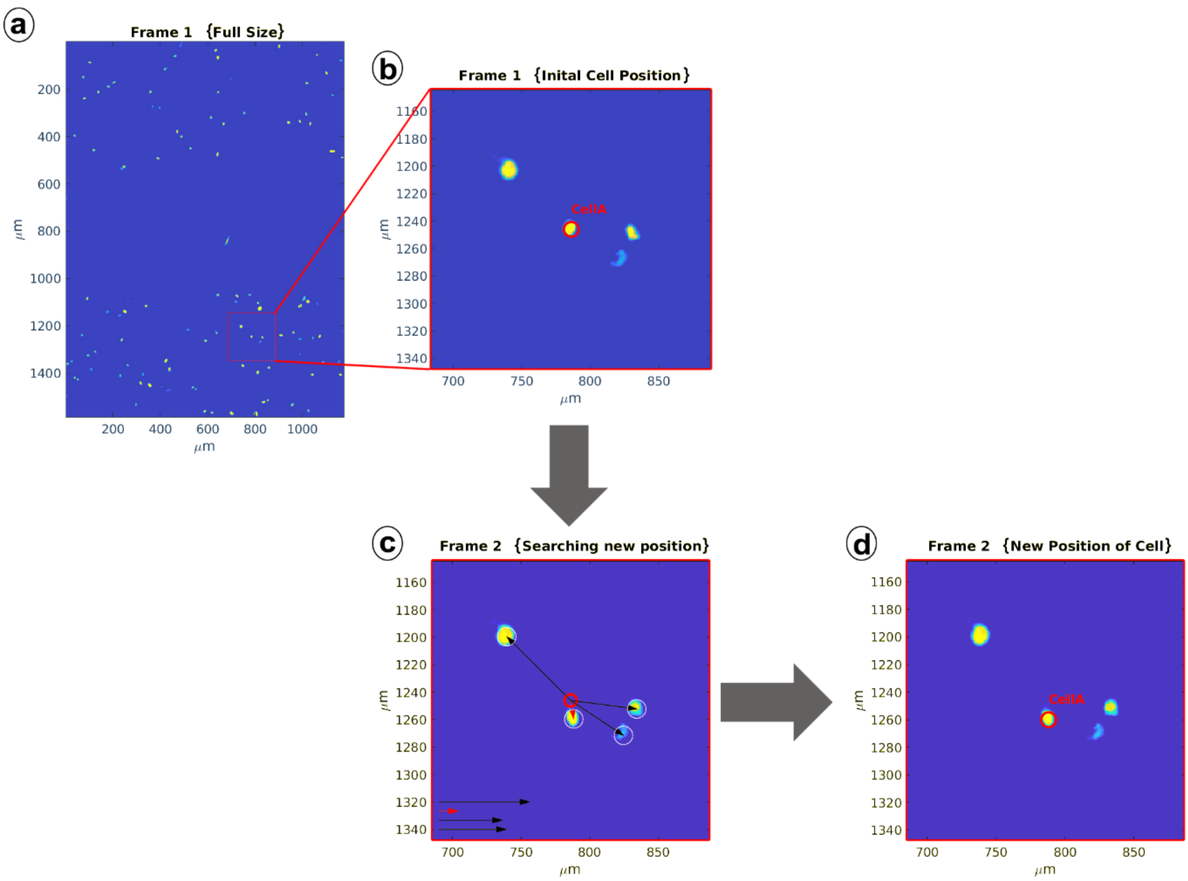

Figure S2. A schematic diagram explaining the tracking paradigm. (a) A full-size view of the first frame of a multi-frame TIFF file. (b) A selected field of the first frame of the multi-frame TIFF file showing nuclei of 4 cells, the red ring refers to the location of the target cell nucleus. (c) Distances between the last position of the target cell nucleus and the position of each nucleus in the second frame, red arrow refers to the shortest distance. (d) new position (red ring) of the tracked cell.

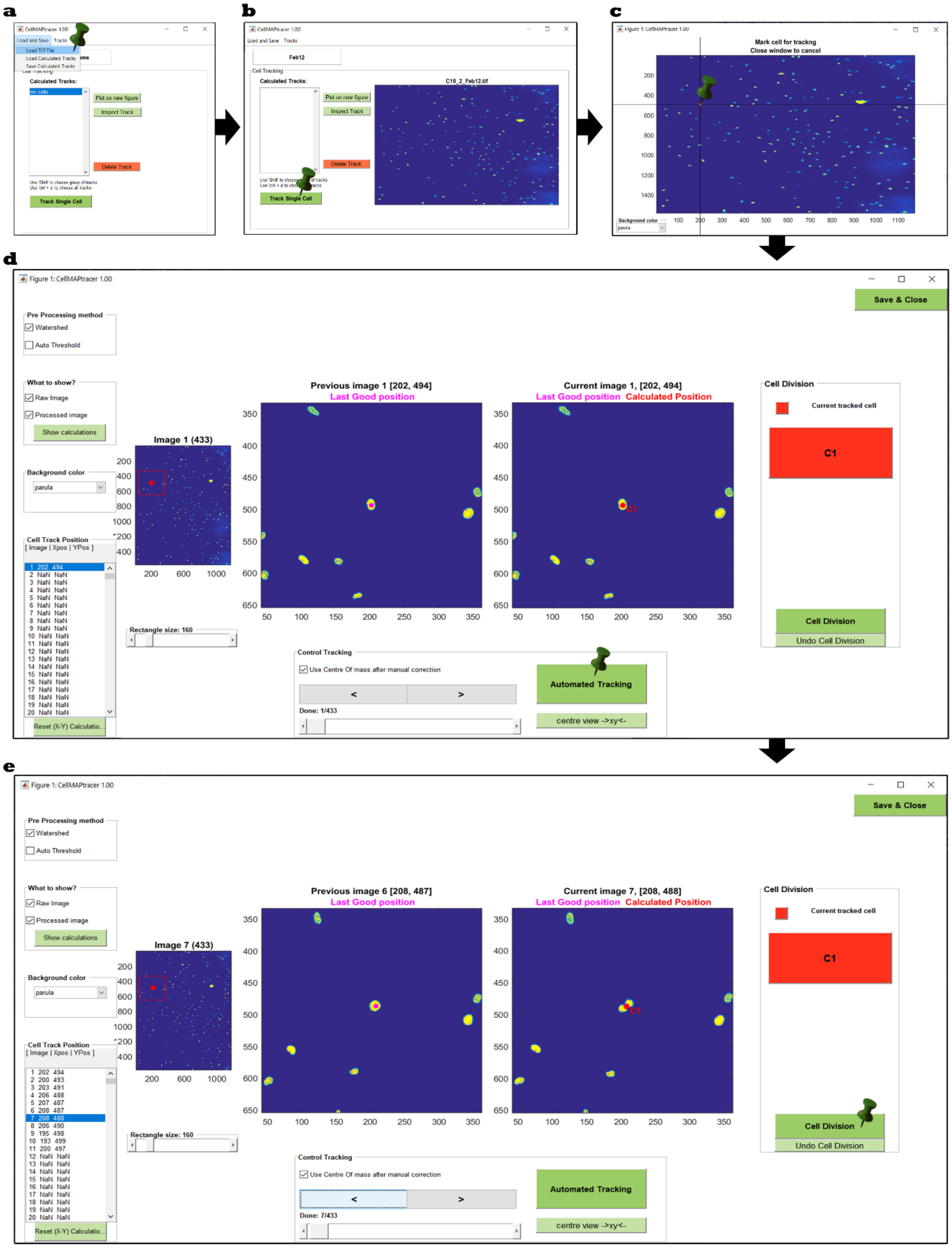

Figure S3. CellMAPtracer workflow. (a) CellMAPtracer front window, where users can load either a TIFF image stack or calculated tracks by clicking on it or nearby. (b) Starting the tracking process by clicking on “Track Single Cell” button. (c) Selecting the cell to be tracked. (d) Tracking window, where users can run the automated tracking and monitor it step-wisely. (e) Marking a cell division by clicking on the “Cell Division” button.

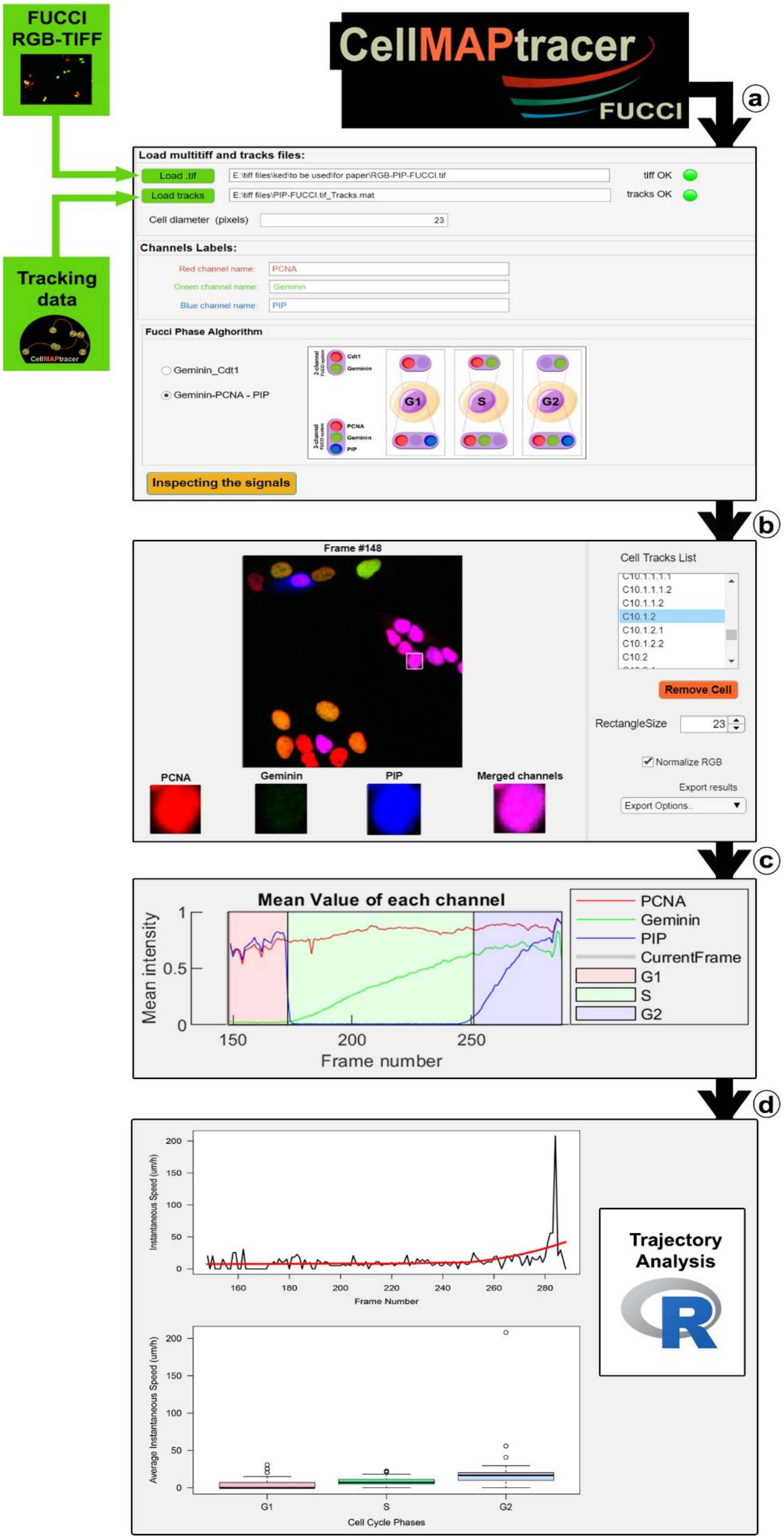

Figure S4. CellMAPtracer FUCCI plug-in workflow. (a) The front window of the FUCCI, plug-in where users need to load the RGB-TIFF image stack and its corresponding tracking data resulted from CellMAPtracer. The desired method for predicting the cell cycle phases should be selected as well. (b) The signals' inspection window, where

users can monitor for each cell the signal of each channel and evaluate the predicted cell cycle phase. (c) An interactive signal plot for cell # 10.1.2 showing the signal profile for each channel across the cell cycle phases. (d) External trajectory analysis showing the speed profile of cell # 10.1.2 across the cell cycle phases. The analysis was done in R based on the attached codes.

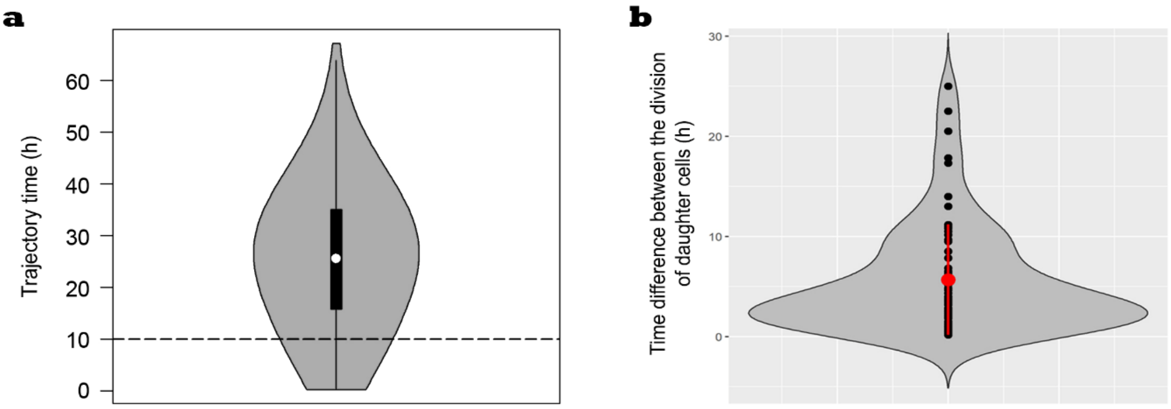

Figure S5. Trajectory time of BT549 daughter cells. (a) Violin plot showing the trajectory time of daughter cells (n = 544), the dashed line refers to the minimum required trajectory time for cells to be included in the heterogeneity analysis between daughter cells. (b) Violin plot showing the time difference between divisions of BT549 daughter cells (n = 71 pairs).

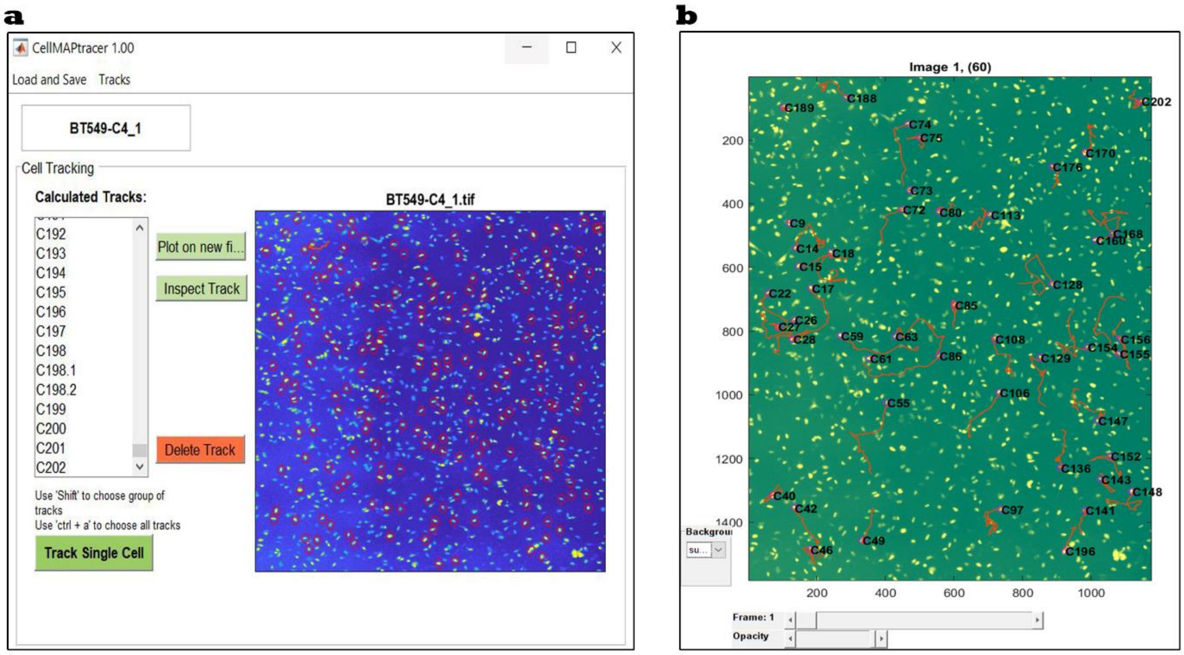

Figure S6. Evaluating the performance and accuracy of CellMAPtracer using, a multi-TIFF stack of dense population of BT549 cells. (a) The main user interface window showing the tracked cells (red circles). (b) Outcome of tracking showing a subset of cells that needed manual correction.

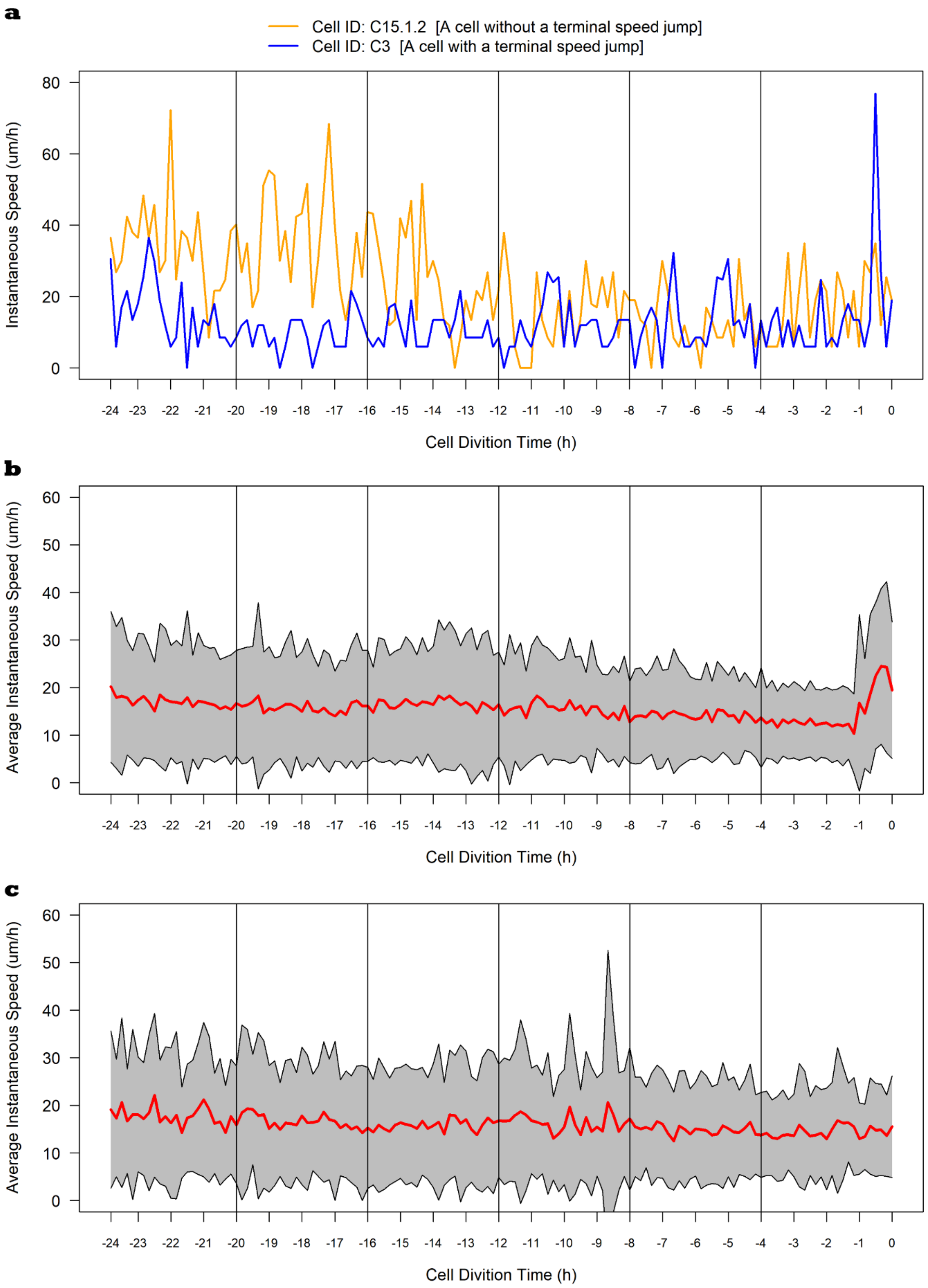

Figure S7. Speed Profile during the preparatory and the G2-M phases. (a) Examples of cells with a terminal speed jump [C3] and without [C15.1.2]. (b) Speed Profile of cells (n = 92) with terminal speed jump. (c) Speed Profile of cells (n = 60) with terminal speed jump. Red line shows the average instantaneous speeds whereas the gray shaded area shows the standard deviation.
