# Supplementary material for: CellMAPtracer: A User-Friendly Tracking Tool for Long-Term Migratory and Proliferating Cells Associated with FUCCI Systems
Source: Cells. 2021 Feb 22;10(2):469. doi: 10.3390/cells10020469 (PMC7927118; doi:10.3390/cells10020469)
Supplement: Supplementary file 1 [file cells-10-00469-s001.zip › CellMAPtracer Supplementary Materials/Supplementary Table S1.pdf]

Supplementary Table S1

CellMAPtracer: A user-friendly tracking tool for long-term migratory and proliferating cells associated with FUCCI systems

Salim Ghannoum[1*], Kamil Antos[2*], Waldir Leoncio Netto[3], Cecil Gomes[4], Alvaro Köhn-Luque[3] and Hesso Farhan[1]

1. Department of Molecular Medicine, Institute of Basic Medical Sciences, University of Oslo, Norway.
2. Department of Integrative Medical Biology, Umeå University, Umeå, Sweden.
3. Oslo Centre for Biostatistics and Epidemiology, Faculty of Medicine, University of Oslo, Norway.
4. University of Arizona Cancer Center, University of Arizona, Tucson, Arizona, USA

* Correspondence: salim.ghannoum@medisin.uio.no; kamil.antos01@umu.se; Tel.: +46 76 5770129 (S.G.)

Supplementary Table S1: A comparative analysis of CellMAPtracer against other tracking tools.

Tool Name	Availability	Platform	Source code	Tracking	Cell division	Inspectability	Correctability	Categorized outcome	FUCCI
Braincells [1]	Free	Win	No	Auto	No	No	No	No	No
CellMAPtracer [2]	Free	Win/Lin/Mac/Matlab	Yes	Semi	Yes	Yes	Yes	Yes	Yes
CellProfiler [3]	Free	Win/Lin/Mac	Yes	Auto	No	No	No	No	No
CellTrack [4]	Free	Win/Lin/Mac	No	Auto	No	Limited	Limited	No	No
CellTracker [5]	Free	Win	No	Semi	No	Limited	Limited	No	No
ClusterTrack [6]	Free	Matlab	Yes	Auto	No	No	No	No	No
DcellIQ [7]	Free	Matlab	Yes	Auto	Yes	No	No	No	No
DeepCell [8]	Free	Python/ImageJ	Yes	Auto	Yes	Yes	Yes	No	No
DeepTree [9]	Free	Python/ImageJ	Yes	Auto	Yes	Yes	Yes	No	No
DIAS [10]	Paid	Win/Mac	No	Auto	No	No	No	No	No
DYNAMIK [11]	Free	Matlab	Yes	Auto	Limited	No	No	No	No
FastTracks [12]	Free	Win	Yes	Auto	No	No	No	No	No
FUCCIJ [13]	Free	ImageJ	No	Auto	Yes	Yes	Yes	No	Yes
ICY [14]	Free	Java	Yes	Auto	No	No	No	No	No
ImarisTrack [15]	Paid	Win/Mac	No	Auto	Limited	Yes	Yes	No	No
LevelSetTracker [16]	Free	Matlab	Yes	Auto	No	Limited	Limited	No	No
LineageTracker [17]	Free	ImageJ	No	Auto	Yes	No	No	No	No
ManualTracking [18]	Free	ImageJ	Yes	Manual	Limited	Limited	Limited	No	No
MetaMorph [19]	Paid	Win	No	Auto	No	No	No	No	No
MTrackJ [20]	Free	ImageJ	Yes	Manual	No	No	No	No	No
NucliTrack [21]	Free	Win/Lin/Mac	Yes	Auto	Yes	Yes	Yes	No	No
ParticleTracker [22]	Free	ImageJ	Yes	Auto	No	No	No	No	No
QuimP [23]	Free	ImageJ	No	Auto	No	No	No	No	No
TLA [24]	Free	Matlab	Yes	Auto	No	No	No	No	No
TrackMate [25] + StarDist [26]	Free	Python/Fiji	Yes	Auto	Yes	Yes	Yes	No	No
TrackMate [25] + FastFUCCI [27]	Free	Fiji	Yes	Auto	Yes	Yes	Yes	No	Yes
tTt [28]	Free	Win	Yes	Semi	Yes	Yes	Yes	No	No

Refrences

1. Hand, A.; Sun, T.; Barber, D.; Hose, D.; MacNeil, S. Automated tracking of migrating cells in phase-contrast video microscopy sequences using image registration. *Journal of microscopy* **2009**, *234*, 62-79.
2. Antos, K.; Ghannoum, S. CellMAPtracer 1.1 (Version v1.1). Zenodo. http://doi.org/10.5281/zenodo.4319619. **2020**.

3. Carpenter, A.E.; Jones, T.R.; Lamprecht, M.R.; Clarke, C.; Kang, I.H.; Friman, O.; Guertin, D.A.; Chang, J.H.; Lindquist, R.A.; Moffat, J. CellProfiler: image analysis software for identifying and quantifying cell phenotypes. *Genome biology* **2006**, *7*, 1-11.
4. Sacan, A.; Ferhatosmanoglu, H.; Coskun, H. CellTrack: an open-source software for cell tracking and motility analysis. *Bioinformatics* **2008**, *24*, 1647-1649.
5. Shen, H.; Nelson, G.; Kennedy, S.; Nelson, D.; Johnson, J.; Spiller, D.; White, M.R.; Kell, D.B. Automatic tracking of biological cells and compartments using particle filters and active contours. *Chemometrics and Intelligent Laboratory Systems* **2006**, *82*, 276-282.
6. Matov, A.; Applegate, K.; Kumar, P.; Thoma, C.; Krek, W.; Danuser, G.; Wittmann, T. Analysis of microtubule dynamic instability using a plus-end growth marker. *Nature methods* **2010**, *7*, 761-768.
7. Li, F.; Zhou, X.; Ma, J.; Wong, S.T. Multiple nuclei tracking using integer programming for quantitative cancer cell cycle analysis. *IEEE transactions on medical imaging* **2009**, *29*, 96-105.
8. Moen, E.; Borba, E.; Miller, G.; Schwartz, M.; Bannon, D.; Koe, N.; Camplisson, I.; Kyme, D.; Pavelchek, C.; Price, T. Accurate cell tracking and lineage construction in live-cell imaging experiments with deep learning. **2019**.
9. Ulicna, K.; Vallardi, G.; Charras, G.; Lowe, A.R. Automated deep lineage tree analysis using a Bayesian single cell tracking approach. *bioRxiv* **2020**.
10. Wessels, D.; Kuhl, S.; Soll, D.R. Application of 2D and 3D DIAS to motion analysis of live cells in transmission and confocal microscopy imaging. In *Dictyostelium discoideum Protocols*, Springer: 2006; pp. 261-279.
11. Mosig, A.; Jäger, S.; Wang, C.; Nath, S.; Ersoy, I.; Palaniappan, K.-p.; Chen, S.-S. Tracking cells in life cell imaging videos using topological alignments. *Algorithms for Molecular Biology* **2009**, *4*, 1-9.
12. DuChez, B.J. Automated tracking of cell migration with rapid data analysis. *Current protocols in cell biology* **2018**, *76*, 12.12. 11-12.12. 16.
13. Roccio, M.; Schmitter, D.; Knobloch, M.; Okawa, Y.; Sage, D.; Lutolf, M.P. Predicting stem cell fate changes by differential cell cycle progression patterns. *Development* **2013**, *140*, 459-470.
14. de Chaumont, F.; Dallongeville, S.; Olivo-Marin, J.-C. ICY: A new open-source community image processing software. In Proceedings of 2011 IEEE International Symposium on Biomedical Imaging: From Nano to Macro; pp. 234-237.
15. OMICS_06586. ImarisTrack. Availabe online: http://www.bitplane.com/imaris/imaristrack (accessed on 31/1/2021).
16. Dzyubachyk, O.; Essers, J.; Cappellen, W.A.v.; Baldeyron, C.; Inagaki, A.; Niessen, W.J.; Meijering, E. Automated analysis of time-lapse fluorescence microscopy images: from live cell images to intracellular foci. *Bioinformatics* **2010**, *26*, 2424-2430.
17. Downey, M.; Vance, K.W.; Bretschneider, T. Lineagetracker: A statistical scoring method for tracking cell lineages in large cell populations with low temporal resolution. In Proceedings of 2011 IEEE International Symposium on Biomedical Imaging: From Nano to Macro; pp. 1913-1916.
18. Cordelières, F.P. Manual tracking. *Institut Curie, Orsay (France)* **2005**.
19. Corp, M.D. Metamorph software, Availabe online: http://www.moleculardevices.com (accessed on 31/1/2021).
20. Meijering, E.; Dzyubachyk, O.; Smal, I. Methods for cell and particle tracking. In *Methods in enzymology*, Elsevier: 2012; Vol. 504, pp. 183-200.
21. Cooper, S.; Barr, A.R.; Glen, R.; Bakal, C. NucliTrack: an integrated nuclei tracking application. *Bioinformatics* **2017**, *33*, 3320-3322.
22. Sbalzarini, I.F.; Koumoutsakos, P. Feature point tracking and trajectory analysis for video imaging in cell biology. *Journal of structural biology* **2005**, *151*, 182-195.

23. Bosgraaf, L.; Van Haastert, P.J.; Bretschneider, T. Analysis of cell movement by simultaneous quantification of local membrane displacement and fluorescent intensities using Quimp2. *Cell motility and the cytoskeleton* **2009**, *66*, 156-165.
24. Huth, J.; Buchholz, M.; Kraus, J.M.; Schmucker, M.; Von Wichert, G.; Krndija, D.; Seufferlein, T.; Gress, T.M.; Kestler, H.A. Significantly improved precision of cell migration analysis in time-lapse video microscopy through use of a fully automated tracking system. *BMC cell biology* **2010**, *11*, 1-12.
25. Tinevez, J.-Y.; Perry, N.; Schindelin, J.; Hoopes, G.M.; Reynolds, G.D.; Laplantine, E.; Bednarek, S.Y.; Shorte, S.L.; Eliceiri, K.W. TrackMate: An open and extensible platform for single-particle tracking. *Methods* **2017**, *115*, 80-90.
26. Fazeli, E.; Roy, N.H.; Follain, G.; Laine, R.F.; von Chamier, L.; Hänninen, P.E.; Eriksson, J.E.; Tinevez, J.-Y.; Jacquemet, G. Automated cell tracking using StarDist and TrackMate. *F1000Research* **2020**, *9*.
27. Koh, S.-B.; Mascalchi, P.; Rodriguez, E.; Lin, Y.; Jodrell, D.I.; Richards, F.M.; Lyons, S.K. A quantitative FastFUCCI assay defines cell cycle dynamics at a single-cell level. *Journal of cell science* **2017**, *130*, 512-520.
28. Hilsenbeck, O.; Schwarzfischer, M.; Skylaki, S.; Schauberger, B.; Hoppe, P.S.; Loeffler, D.; Kokkaliaris, K.D.; Hastreiter, S.; Skylaki, E.; Filipczyk, A. Software tools for single-cell tracking and quantification of cellular and molecular properties. *Nature biotechnology* **2016**, *34*, 703-706.
